# Serum Indicators of Oxidative Damage from Embedded Metal Fragments in a Rat Model

**DOI:** 10.1155/2022/5394303

**Published:** 2022-02-02

**Authors:** John F. Kalinich, Vernieda B. Vergara, Jessica F. Hoffman

**Affiliations:** Internal Contamination and Metal Toxicity Program, Armed Forces Radiobiology Research Institute, Uniformed Services University, Bethesda, Maryland 20889-5648, USA

## Abstract

Injuries suffered in armed conflicts often result in embedded metal fragments. Standard surgical guidance recommends leaving embedded fragments in place except under certain circumstances in an attempt to avoid the potential morbidity that extensive surgery often brings. However, technological advances in weapon systems and insurgent use of improvised explosive devices now mean that practically any metal can be found in these types of wounds. Unfortunately, in many cases, the long-term toxicological properties of embedded metals are not known, further complicating treatment decisions. Because of concerns over embedded metal fragment injuries, the U.S. Departments of Defense and Veterans' Affairs developed a list of “metals of concern” for these types of injuries. In this study, we selected eight of these metals including tungsten, nickel, cobalt, iron, copper, aluminum, lead, and depleted uranium to investigate the long-term health effects using a rodent model developed in our Institute to study embedded fragment injuries. In this report, we show that metals surgically implanted into the gastrocnemius muscle of laboratory rats to simulate a shrapnel wound induce a variety of cytokines including IFN-*γ*, IL-4, IL-5, IL-6, IL-10, and IL-13. TNF-*α* and KC/GRO were not affected, and IL-1*β* was below the limit of detection. Serum levels of C-reactive protein were also affected, increasing with some metals and decreasing with others. The TBARS assay, an assessment of lipid peroxidation, demonstrated that implanted aluminum and lead increased markers of lipid peroxidation in serum. Taken together, the results suggest that serum cytokine levels, as well as other indicators of oxidative damage, may prove useful in identifying potential adverse health effects of embedded metals.

## 1. Introduction

Metals can be internalized by several routes including ingestion and inhalation and through wounds. The health effects of inhalation and ingestion of metals have been well studied; however, the effects of embedded metal fragments have received less attention. One of the reasons for this was the long-time assumption that embedded metals, especially those from war wounds, were biologically inert [1]. Occasional reports in the scientific literature describing the health effects of metal fragment wounds suffered in battle many years prior suggested that this was not always the case [2–8]. Because of the risk of morbidity that excessive surgery can bring, standard surgical guidance is to leave embedded fragments in place unless they are easily accessible or may present a life-threatening health issue in the future [9]. Technological improvements in armor protection and advancements in battlefield medicine now mean that injuries that were once inherently fatal are now survivable. However, this results in a cohort of individuals that now carry retained metal fragments potentially for the rest of their lives. The extent of the issue was made clear by the U.S. Department of Defense which estimated that there are over 40,000 U.S. military personnel with retained metal fragments as a result of their service in the Iraq and Afghanistan conflicts [10]. In many cases, the toxicological and carcinogenic properties of these metals are not known. As a result, the U.S. Departments of Defense (DoD) and Veterans Affairs (DVA) developed a list of military-relevant “metals of concern” with respect to embedded metal fragments [11, 12] with the intent of identifying those individuals with embedded fragments, the metal or metals retained, and their follow-up long-term health care.

Adverse health effects due to embedded metals are not limited to war injuries. Published case reports also described workplace injuries with embedded metal fragments from lawnmower and chainsaw blades that over time resulted in granuloma formation [13, 14]. The failure of implanted medical and prosthetic devices such as artificial knees and metal-on-metal hip replacements has also occasionally resulted in health issues [15, 16]. Often times, additional surgery is required to replace the device although there have been cases where the device failure has resulted in the death of the patient [17].

The ability to identify impending health risks as a result of embedded metals is limited. As noted in the above citations, most of the adverse health effects induced by embedded metals from battlefield wounds were recognized only when a tumor or granuloma formed. A protocol by which potential adverse health outcomes could be identified early enough so that treatment plans, including more extensive surgical removal of the embedded fragment, could be initiated would be useful. While biomarkers of adverse health issues have not been thoroughly investigated with respect to embedded metal fragments of military-relevant metals, there are numerous reports in the literature on the induction of cytokines and indicators of oxidative stress in patients with failed implanted medical devices, most notably, metal-on-metal hip replacements. For example, Christiansen et al. reported on the cytokine profile of patients with aseptic loosening of hip replacements [18]. In their study, the proinflammatory cytokines IL-1*β*, IL-2, IL-8, IFN-*γ*, and TNF-*α* were significantly increased as was the anti-inflammatory cytokine, IL-10. Similar results were seen for patients with metal-on-polyethylene hip replacements, as well as those with total knee replacements [19, 20]. Changes in cytokine expression and the induction of oxidative damage to DNA, lipids, and proteins were also seen in metal exposure via inhalation [21]. Taken together, these results suggest that a battery of assessments, both cytokine levels and oxidative damage markers, might be useful in detecting early adverse changes induced by embedded fragments of military-relevant metals.

Using a rodent model developed in our Institute to study the health effects of embedded metal fragments, we investigated the effect of 8 metals chosen from the DoD and DVA list of “metals of concern” on cytokine levels and indicators of oxidative stress in the serum of laboratory rats implanted with metals for up to 12 months. This research is part of a larger collaborative effort with the Department of Veterans' Affairs Medical Center in Baltimore, Maryland, the University of Maryland School of Medicine, and the University of Kentucky to study the potential health effects of embedded metals in our rodent model alongside an expanded human study with military service personnel with retained metal fragments.

## 2. Materials and Methods

### 2.1. Animals and Animal Husbandry

The Armed Forces Radiobiology Research Institute (AFRRI) Institutional Animal Care and Use Committee (IACUC) approved all animal use prior to initiation under protocol number 2016-05-006. All procedures were conducted in compliance with the *Guide for the Care and Use of Laboratory Animals* [22] in an Association for Assessment and Accreditation of Laboratory Animal Care- (AAALAC-) accredited facility. Two hundred and eighty-eight male Sprague-Dawley (*Rattus norvegicus*) rats, approximately 30 days old and weighing 75-100 g, were obtained from Envigo (Barrier 208A, Frederick, MD, USA). After arriving at the vivarium, animals were allowed to acclimate for at least two weeks. Rats were pair-housed throughout the study in plastic microisolator cages with filter tops. Teklab Sani-Chips (Envigo) were used as bedding and changed 2-3 times per week. Vivarium rooms were maintained at 21 ± 2°C with 30-70% humidity. A 12 : 12 h light : dark cycle was maintained with lights on at 0600. Rats were fed a standard rodent chow (Teklad Global Rodent Diet 8604, Envigo) with water available *ad libitum*.

### 2.2. Experimental Design

Our Institute previously developed a rodent model to study the health effects of embedded metal fragments, such as those suffered in a shrapnel wound [23]. In this study, we used that model to investigate the effects of eight military-relevant metals including tungsten (W), nickel (Ni), cobalt (Co), iron (Fe), copper (Cu), aluminum (Al), lead (Pb), and depleted uranium (DU). Tantalum (Ta) was used as a control for any changes resulting from the surgical procedure or due to the presence of a foreign material in the muscle. Tantalum is considered inert and has been used for implanted prosthetic devices [24–26]. Earlier investigations have shown no differences between naïve and tantalum-implanted rats [27–29]. Thus, the total number of rats needed for the study could be reduced and the ARRIVE guidelines met [30]. The rats were randomly assigned to one of the nine metal implantation groups with *n* = 8 per metal. In the metal groups, surgery was conducted in 4 different cohorts: 1-, 3-, 6-, or 12-month postimplantation surgery (i.e., “time from implant”). A total of 288 rats (n = 8 × 9 metal groups × 4 cohorts) were used in the study.

### 2.3. Metal Pellets

Metal pellets for implantation were obtained from Alfa Aesar (Ward Hill, MA, USA) with the exception of DU which was purchased from Aerojet Ordnance (Jonesboro, TN, USA). All pellets were cylinders 1 mm in diameter by 2 mm in length. Prior to implantation, pellets were cleaned and chemically sterilized as previously described [31].

### 2.4. Metal Pellet Implantation Surgery

Metal pellets were surgically implanted bilaterally in the gastrocnemius muscle of rats as previously described [32, 33]. Briefly, animals were initially anesthetized using isoflurane (Baxter Healthcare, Deerfield, IL, USA) in an induction chamber. Anesthesia was then maintained throughout the surgical period using a nose cone with a scavenger/recapture system. The surgical site was clipped, swabbed with 70% isopropanol, and cleansed with betadine (Purdue Pharma LP, Stamford, CT, USA). Prior to surgery, a prophylactic dose of buprenorphine (0.05-0.1 mg/kg, s.c., Reckitt and Colman, Hull, UK) was administered to serve as an analgesic. For animal identification purposes, two methods were employed. A small transponder (Electronic Lab Animal Monitoring System, Bio-Medic Data Systems, Seaford, DE, USA) was injected subcutaneously in the middorsal thoracic region. The transponders were programmed with a unique animal identification number that can be read with a low-power radio frequency scanner. An ear punch system was used as the second backup identification system in the event of transponder failure. For pellet implantation, using an aseptic technique, a small incision approximately 5 mm in length was made through the skin of each hind leg to expose the gastrocnemius muscle. Each gastrocnemius muscle was implanted with two sterile pellets spaced approximately 1.5 mm apart on the lateral side of the muscle by placing the sterile pellet in a 16-gauge needle. Using a specially designed plunger placed inside the needle, the needle was inserted into the gastrocnemius and the plunger depressed forcing the pellet into the muscle. Tissue adhesive (VetBond; 3M Corporation, St. Paul, MN, USA) was used to seal the incision. After surgery, rats were closely monitored until ambulatory. The surgery sites were examined daily for two weeks to assess for signs of inflammation, infection, and local metal toxicity and, after that time, weekly for the duration of the study.

### 2.5. Euthanasia and Sample Collection

Upon reaching their experimental endpoint or when indicated by guidelines approved by the IACUC, rats were deeply anesthetized using isoflurane and blood was collected for hematological analysis as well as for serum isolation. Following blood collection, the rats were humanely euthanized under deep isoflurane by exsanguination and confirmatory pneumothorax as per the guidelines of the American Veterinary Medical Association [34]. A complete gross pathology examination was conducted, and a variety of tissues were collected for further examination as has been described in earlier publications [32, 33].

### 2.6. Serum Preparation

Blood was collected in serum separator tubes (Becton-Dickinson, Franklin Lakes, NJ, USA), and the tubes were inverted 5 times and then allowed to sit undisturbed for 30 min at room temperature. The tubes were centrifuged at 1200 × *g* for 10 min at room temperature. The serum was aliquoted into 1.5 ml centrifuge tubes and stored at -80°C until analyzed.

### 2.7. Serum Proinflammatory Markers

Serum levels of interferon (IFN-) *γ*, interleukin- (IL-) 1*β*, IL-4, IL-5, IL-6, KC/GRO, IL-10, IL-13, and tumor necrosis factor- (TNF-) *α* were determined using the rat serum proinflammatory panel-2 kit from Meso Scale Discovery (kit # K15059D, Rockville, MD, USA). The technique uses a special electrode-containing 96-well plate coated with the appropriate capture antibodies to bind the analytes of interest. Addition of detection antibodies conjugated to a proprietary chemiluminescence tag allows quantitation of the targeted proteins using a Meso Scale Discovery SQ120 QuickPlex Reader. The instrument applies a voltage to the electrodes in the plate that causes the tagged detection antibodies to emit light. The intensity of the light provides a quantitative measure of the analytes in the serum sample, with concentrations determined by comparison to a standard curve using the Meso Scale Discovery Workbench software. The reported lower limits of detection of the procedure are as follows: IFN-*γ*: 0.65 pg/ml; IL-1*β*: 4.26 pg/ml; IL-4: 0.52 pg/ml; IL-5: 12.8 pg/ml; IL-6: 3.16 pg/ml; KC/GRO: 0.77 pg/ml; IL-10: 1.42 pg/ml; IL-13: 0.96 pg/ml; and TNF-*α*: 0.23 pg/ml.

### 2.8. C-reactive Protein Assay

Serum levels of C-reactive protein were determined using the Rat C-Reactive Protein ELISA kit (kit #ab108827, Abcam, Cambridge, MA, USA). Briefly, serum samples were diluted to 1 : 60,000 with the sample diluent provided in the kit. Samples and standards (50 *μ*l/well) were added to a 96-well antibody-coated plate and incubated at room temperature with shaking (250 rpm, Incubating Microplate Shaker, VWR, Radnor, PA, USA) for 2 h. After washing, a biotinylated detector antibody was added and incubation continued for 1 h at room temperature with shaking (250 rpm). The plate was then washed, the streptavidin-peroxidase conjugate was added, and the plate was incubated for 20 min at room temperature with shaking at 250 rpm. After stopping the reaction, the plate was immediately read at 450 nm using a plate reader (BioTek Synergy Model H1M Multimodal Plate Reader with GEN5 Software, BioTek Instruments, Winooski, VT, USA). The concentration of C-reactive protein in the samples was determined by comparing the absorbance readings of the samples to those from a standard curve. The lower limit of detection of the assay is 0.25 ng/ml.

### 2.9. Thiobarbituric Acid Reactive Substance (TBARS) Assay

Lipid peroxidation was monitored using the thiobarbituric acid reactive substance (TBARS) assay kit from Cayman Chemical (kit #700870, Ann Arbor, MI, USA). The TBARS assay measures the amount of malondialdehyde (MDA), a naturally occurring product of lipid peroxidation, in a sample by reacting it with thiobarbituric acid (TBA). The MDA-TBA adduct can then be detected either colorimetrically or fluorometrically. Briefly, 100 *μ*l of sample or standard was placed in a 1.5 ml centrifuge tube; 100 *μ*l of 10% trichloroacetic acid was added followed by 800 *μ*l of “Color Reagent” (TBA in acetic acid/sodium hydroxide, supplied in the kit). After vortexing, the tubes were placed in a 95°C heat block for 60 min and then placed in an ice bath for 10 min. The tubes were centrifuged at 1600 × *g* for 10 min, and 200 *μ*l of the resulting supernatant, in duplicate, was removed and placed in a black plate for fluorometric analysis. The BioTek Synergy Model H1M Multimodal Plate Reader was used with an excitation wavelength of 532 nm and an emission wavelength of 585 nm to assess the concentration of the MDA-TBA adduct in both the standards and the samples. The lower limit of detection of the assay for MDA is 0.01 *μ*M.

### 2.10. Advanced Oxidative Protein Product (AOPP) Assay

Serum samples were analyzed for advanced oxidative protein products using the OxiSelect AOPP Assay Kit (kit # STA-318, Cell Biolabs, Inc., San Diego, CA, USA). Serum samples were diluted to 1 : 20 with the assay diluent included in the kit, and 200 *μ*l of the prepared samples was added per well to a 96-well plate. A chloramine reaction initiator, provided in the kit, was added to each well and the plate incubated for 5 min at room temperature with shaking (250 rpm). The reaction was then terminated and the plate read at 340 nm using the BioTek Synergy Model H1M Multimodal Plate Reader. The AOPP concentrations in the serum samples were determined by comparing to a standard curve prepared using chloramine-T. The lower limit of detection is approximately 1.25 *μ*M.

### 2.11. Glutathione Assay

Glutathione levels in the collected serum samples were determined using the DetectX Glutathione Fluorescent Detection Kit (kit # K006, Arbor Assays, Ann Arbor, MI, USA). Initially, serum samples were diluted with an equal volume of ice-cold 5% 5-sulfo-salicylic acid (catalog # S2130, Sigma Chemical Co., St. Louis, MO, USA) and left on ice for 10 min. Samples were centrifuged for 10 min at 21,000 × *g* after which 30 *μ*l of the resulting supernatant was carefully removed, added to a fresh centrifuge tube, and diluted with Assay Buffer provided in the kit. The final sample dilution was 1 : 25. Diluted samples and standards were added to the plate at 50 *μ*l/well followed by 25 *μ*l of ThioStar™ Diluent provided in the kit. After a 15 min incubation at room temperature, fluorescence was read using the BioTek Synergy Model H1M Multimodal Plate Reader with excitation at 370 nm and emission at 510 nm. This represents the free glutathione concentration. After addition of the kit-provided “Reaction Mixture” (25 *μ*l/well) and a second 15 min room temperature incubation, the plate was again read using the conditions described above. This reading represents the total glutathione concentration. The reported lower limits of detection are 38 nM and 42 nM for the free and total glutathione levels, respectively.

### 2.12. Statistical Analysis

Assay data are expressed as the mean of 8 independent determinations with figure errors shown as standard error of the mean. Cytokine data are given as pg/ml; C-reactive protein data as *μ*g/ml; and TBARS, AOPP, and GSH as *μ*M. Data were statistically analyzed by two-way ANOVA using the variables of implanted metal and time from implant followed by Sidak's multiple comparisons test where each metal implant group within a time from implant is compared back to the corresponding Ta-implanted group value. Analyses were performed using GraphPad Prism Software (version 9.2.0, La Jolla, CA, USA). In all cases, *p* values < 0.05 were considered significant.

## 3. Results

**Figure 1:**
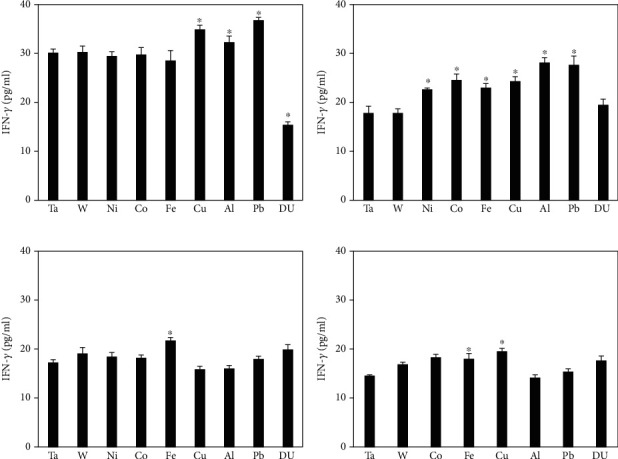
IFN-*γ* levels in serum from metal-implanted rats: (a) 1 month postimplantation; (b) 3 months postimplantation; (c) 6 months postimplantation; (d) 12 months postimplantation. Data represent the mean of 8 independent determinations. Error bars are standard error of the mean. Note: the 12 M Ni cohort was euthanized at approximately 6 M as a result of tumor formation. An ∗ indicates a post hoc test between Ta- and target-metal animals at that time point (*p* < 0.05).

Cytokine levels in the collected rat serum were determined using the Meso Scale Discovery rat proinflammatory marker kit. Cytokines assessed included IFN-*γ*, IL-1*β*, IL-4, IL-5, IL-6, KC/GRO, IL-10, IL-13, and TNF-*α*. Serum IL-1*β* levels were consistently below the limit of detection for all metal implantation groups and thus are not presented here.  Figure 1 shows the level of IFN-*γ* in the serum obtained from rats implanted with metals for 1, 3, 6, and 12 months. Overall, IFN-*γ* levels are highest across the board in the 1-month cohort with the Cu, Al, and Pb groups showing significantly higher IFN-*γ* levels than the control. Conversely, the 1-month DU cohort's IFN-*γ* serum levels are significantly lower than those of the control. At 3 months postimplantation, serum IFN-*γ* levels are also significantly higher in the Ni, Co, and Fe groups as well as still being significantly elevated in the Cu, Al, and Pb groups. At 3 months, levels in the DU group were no different than those in the control. By 6 months postimplantation, the only metal group that had significantly higher IFN-*γ* serum levels was the Fe cohort. A similar situation was observed at 12 months postimplantation with only the Fe and Cu groups demonstrating significantly higher serum IFN-*γ* levels than the control.

**Figure 2:**
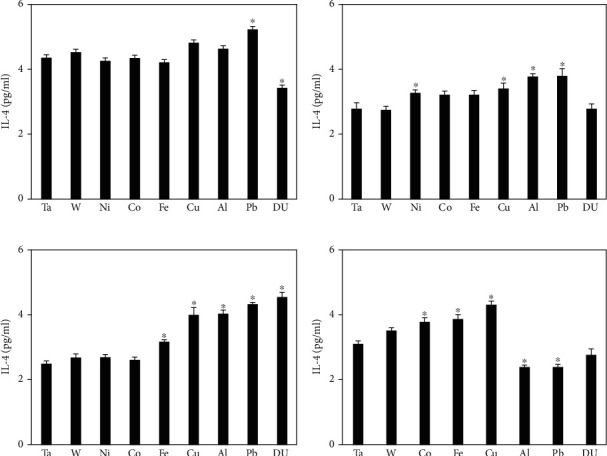
IL-4 levels in serum from metal-implanted rats: (a) 1 month postimplantation; (b) 3 months postimplantation; (c) 6 months postimplantation; (d) 12 months postimplantation. Data represent the mean of 8 independent determinations. Error bars are standard error of the mean. Note: the 12 M Ni cohort was euthanized at approximately 6 M as a result of tumor formation. An ∗ indicates a post hoc test between Ta- and target-metal animals at that time point (*p* < 0.05).

Serum IL-4 levels across the four time points are shown in Figure 2. At 1 month postimplantation, only the Pb and DU groups are significantly different from the control, with Pb being higher and DU lower. At 3 months postimplantation, the DU cohort was no different than the control while the Pb group remained elevated and was joined by the Ni, Cu, and Al groups as well. By 6 months postimplantation, Ni had returned to control levels while Cu, Al, and Pb serum IL-4 levels remained significantly elevated with the Fe and DU groups also showing elevated levels of serum IL-4. By 12 months postimplantation, IL-4 levels remained elevated in the Fe and Cu groups. The Co cohort also demonstrated significantly higher serum IL-4 levels over the control, but the Al and Pb groups were significantly lower than the control while the DU cohort was not significantly different than the control.

**Figure 3:**
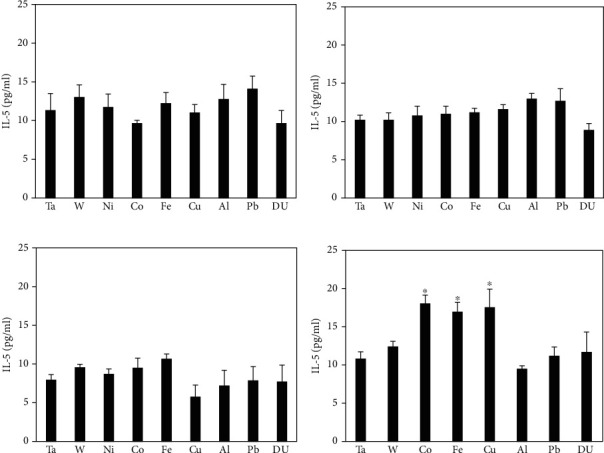
IL-5 levels in serum from metal-implanted rats: (a) 1 month postimplantation; (b) 3 months postimplantation; (c) 6 months postimplantation; (d) 12 months postimplantation. Data represent the mean of 8 independent determinations. Error bars are standard error of the mean. Note: the 12 M Ni cohort was euthanized at approximately 6 M as a result of tumor formation. An ∗ indicates a post hoc test between Ta- and target-metal animals at that time point (*p* < 0.05).

Serum IL-5 levels in the metal-implanted groups showed no significant differences at the 1-, 3-, and 6-month time points (Figure 3). However, at 12 months postimplantation, the Co, Fe, and Cu groups all showed significantly higher serum levels of IL-5 when compared to the control.

**Figure 4:**
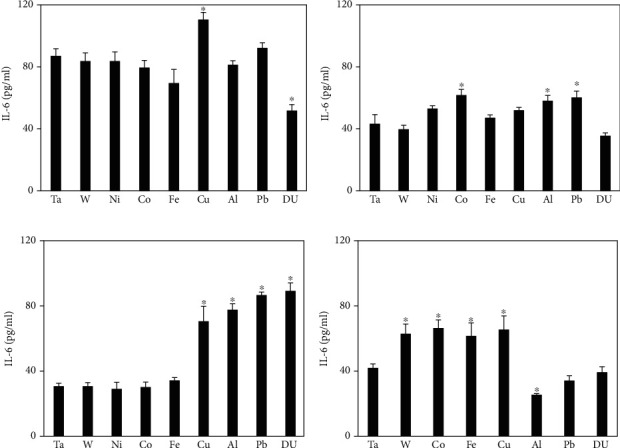
IL-6 levels in serum from metal-implanted rats: (a) 1 month postimplantation; (b) 3 months postimplantation; (c) 6 months postimplantation; (d) 12 months postimplantation. Data represent the mean of 8 independent determinations. Error bars are standard error of the mean. Note: the 12 M Ni cohort was euthanized at approximately 6 M as a result of tumor formation. An ∗ indicates a post hoc test between Ta- and target-metal animals at that time point (*p* < 0.05).

Figure 4 shows IL-6 levels in serum from metal-implanted rats. At 1 month, the Cu group was significantly higher and the DU group significantly lower than the control. By 3 months, both groups were no different than the control, but the Co, Al, and Pb groups showed significantly higher serum levels of IL-6 than the control. At 6 months postimplantation, the Al and Pb groups remained elevated and were joined by the Cu and DU groups while the Co group was not different than the control. The Cu group remained significantly elevated at the 12-month time point as were the W, Co, and Fe groups. Serum IL-6 levels in the Pb and DU groups were not significantly different from those in the control, while levels in the Al were significantly lower.

**Figure 5:**
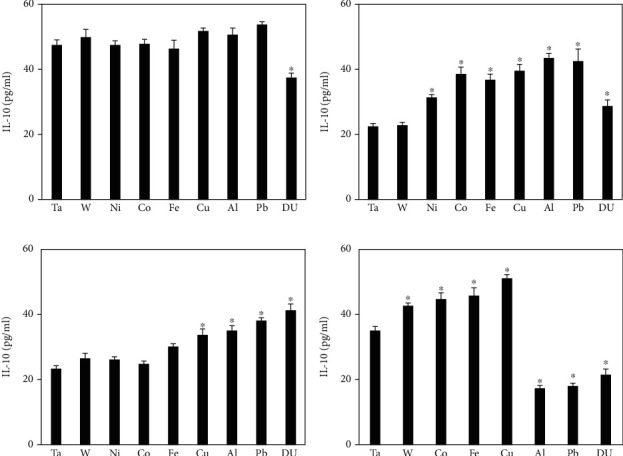
IL-10 levels in serum from metal-implanted rats: (a) 1 month postimplantation; (b) 3 months postimplantation; (c) 6 months postimplantation; (d) 12 months postimplantation. Data represent the mean of 8 independent determinations. Error bars are standard error of the mean. Note: the 12 M Ni cohort was euthanized at approximately 6 M as a result of tumor formation. An ∗ indicates a post hoc test between Ta- and target-metal animals at that time point (*p* < 0.05).

Serum IL-10 levels from the metal-implanted rats are shown in Figure 5. At 1 month postimplantation, there were no significant differences from the control except for the DU group which was significantly lower. However, at the 3-month postimplantation time point, all metal groups showed significantly higher serum IL-10 levels compared to the control, with the exception of the W group. All metal groups at the 12-month postimplantation time point were significantly different than the control with W, Co, Fe, and Cu significantly higher and Al, Pb, and DU significantly lower.

**Figure 6:**
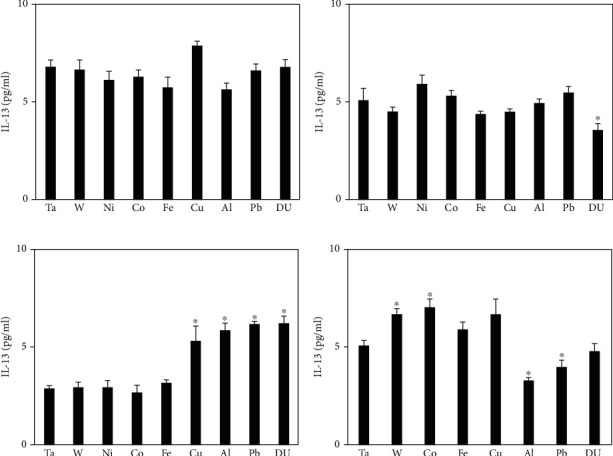
IL-13 levels in serum from metal-implanted rats: (a) 1 month postimplantation; (b) 3 months postimplantation; (c) 6 months postimplantation; (d) 12 months postimplantation. Data represent the mean of 8 independent determinations. Error bars are standard error of the mean. Note: the 12 M Ni cohort was euthanized at approximately 6 M as a result of tumor formation. An ∗ indicates a post hoc test between Ta- and target-metal animals at that time point (*p* < 0.05).

Figure 6 shows serum IL-13 levels in metal-implanted rats. No significant differences were observed at the 1-month postimplantation time point, and only the DU group was significantly lower than the control at the 3-month time point. However, by 6 months postimplantation, the Cu, Al, Pb, and DU groups showed significantly elevated serum IL-13 levels compared to the control. By 12 months postimplantation, these four groups were either no different than the control (Cu, DU) or significantly lower (Al, Pb). Conversely, the W and Co groups demonstrated significantly elevated serum IL-13 levels at this time point.

**Figure 7:**
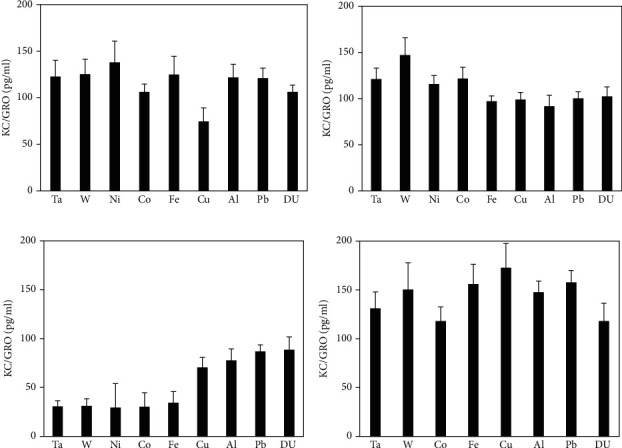
KC/GRO levels in serum from metal-implanted rats: (a) 1 month postimplantation; (b) 3 months postimplantation; (c) 6 months postimplantation; (d) 12 months postimplantation. Data represent the mean of 8 independent determinations. Error bars are standard error of the mean. Note: the 12 M Ni cohort was euthanized at approximately 6 M as a result of tumor formation. An ∗ indicates a post hoc test between Ta- and target-metal animals at that time point (*p* < 0.05).

**Figure 8:**
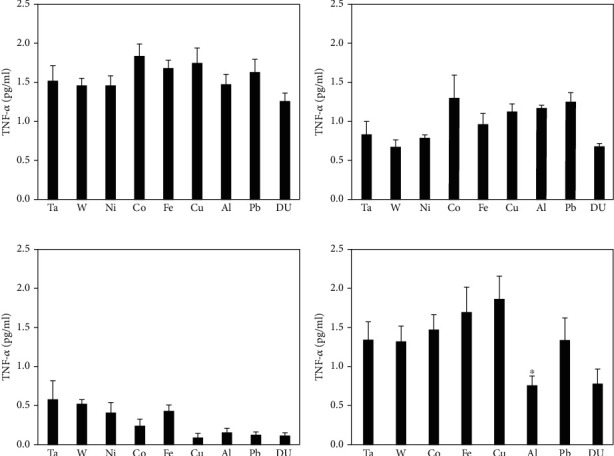
TNF-*α* levels in serum from metal-implanted rats: (a) 1 month postimplantation; (b) 3 months postimplantation; (c) 6 months postimplantation; (d) 12 months postimplantation. Data represent the mean of 8 independent determinations. Error bars are standard error of the mean. Note: the 12 M Ni cohort was euthanized at approximately 6 M as a result of tumor formation. An ∗ indicates a post hoc test between Ta- and target-metal animals at that time point (*p* < 0.05).

Serum levels of KC/GRO and TNF-*α* in metal-implanted rats are shown in Figures 7 and 8, respectively. No significant differences from the control were seen in any of the metal groups at any time points for KC/GRO, while only the 12-month Al group was significantly different from the control with respect to serum TNF-*α* levels.

**Figure 9:**
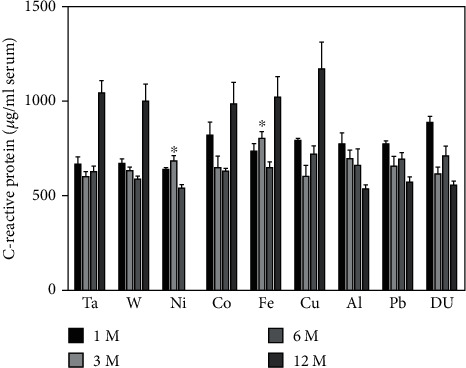
C-reactive protein levels in serum from metal-implanted rats. Data represent the mean of 8 independent determinations. Error bars are standard error of the mean. Note: the 12 M Ni cohort was euthanized at approximately 6 M as a result of tumor formation. An ∗ indicates a post hoc test between Ta- and target-metal animals at that time point (*p* < 0.05).

Figure 9 shows the serum levels of C-reactive protein in the metal-implanted groups. Only the 1-month DU and 3-month nickel and iron groups exhibited serum C-reactive protein levels significantly higher than the control. However, the 12-month postimplantation Al, Pb, and DU groups showed significantly lower serum C-reactive protein as compared to the controls. In fact, the serum levels of C-reactive protein in those groups tended to drop over time while the levels in the other metal-implanted cohorts tended to rise as the animals aged.

**Figure 10:**
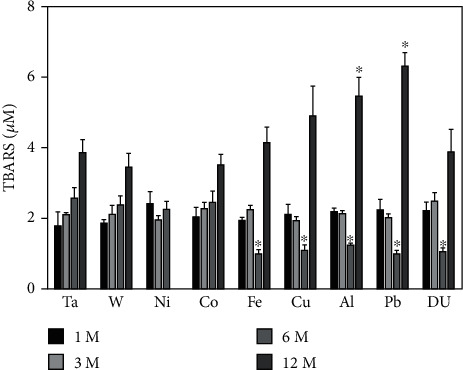
Assessment of lipid peroxidation in serum from metal-implanted rats. Data represent the mean of 8 independent determinations. Error bars are standard error of the mean. Note: the 12 M Ni cohort was euthanized at approximately 6 M as a result of tumor formation. An ∗ indicates a post hoc test between Ta- and target-metal animals at that time point (*p* < 0.05).

The TBARS assay was used as an indicator for lipid peroxidation in this study. As seen in Figure 10, in most of the groups, lipid peroxidation rose over time; however, only the 12-month Al and Pb groups were shown to be statistically greater than the control. Somewhat surprisingly, before the TBARS increase observed at 12 months, the 6-month levels in the Fe, Cu, Al, Pb, and DU groups were significantly lower than those in the control at the same time point.

**Figure 11:**
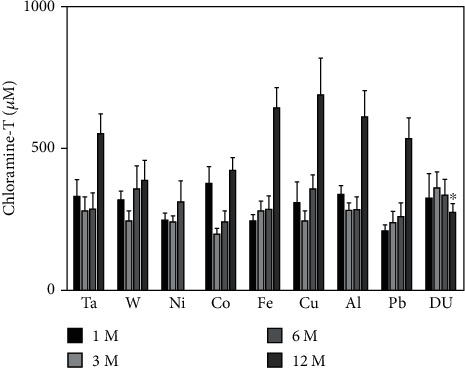
Advanced oxidation protein products in serum from metal-implanted rats. Data represent the mean of 8 independent determinations. Error bars are standard error of the mean. Note: the 12 M Ni cohort was euthanized at approximately 6 M as a result of tumor formation. An ∗ indicates a post hoc test between Ta- and target-metal animals at that time point (*p* < 0.05).

Oxidative protein damage was assessed with the advanced oxidation protein products assay with the results shown in Figure 11. Similar to what was seen with lipid peroxidation, protein oxidation generally increased in all groups over time with the only exception being the 12-month DU group which was significantly lower than the control.

**Figure 12:**
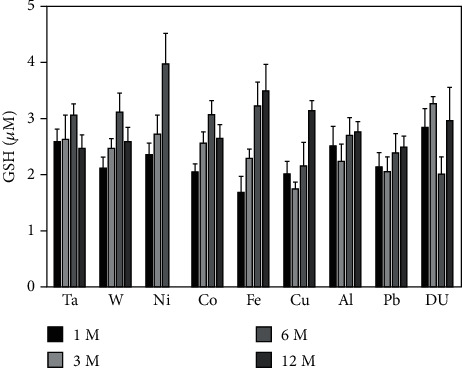
Glutathione levels in serum from metal-implanted rats. Data represent the mean of 8 independent determinations. Error bars are standard error of the mean. Note: the 12 M Ni cohort was euthanized at approximately 6 M as a result of tumor formation. An ∗ indicates a post hoc test between Ta- and target-metal animals at that time point (*p* < 0.05).

In light of the implanted metal effects on lipid peroxidation products found in the serum, we also measured the total glutathione levels in the serum to determine if metal implantation had any effect on antioxidant capacity. As seen in Figure 12, there were no significant differences between any on the metal implantation groups and control at any of the time points assessed.

## 4. Discussion

The ability to identify potential adverse health effects resulting from embedded metals in the body is an area of increasing urgency. With an estimated 40,000 U.S. military personnel with retained metal fragments from the conflicts in Iraq and Afghanistan, as well as an untold number of civilian casualties, the capability to detect early changes as a result of embedded metals is the key to initiating prompt medical intervention strategies, including more extensive surgical removal of the embedded metal if warranted. As a part of our larger multi-institute study on the health effects of embedded fragments, we report here our investigations on cytokine and oxidative damage markers in the serum of laboratory rats implanted with pellets of military-relevant metals. We have previously reported that, with the exception of rhabdomyosarcoma formation in both the Ni- and Co-implanted groups, no metal-induced damage was observed by histopathological examination of the gastrocnemius by a board-certified veterinary pathologist [35]. Rhabdomyosarcoma induction by embedded Ni pellets necessitated early euthanasia of the 12-month Ni cohort based on IACUC criteria while tumor formation in the Co-implanted rats did not reach this level of severity and such were not euthanized early. Another unexpected finding previously reported was the migration and expulsion of the Cu pellets through the skin over the gastrocnemius shortly after 3 months of implantation [35]. Again, no noticeable muscle damage was observed via histopathology assessment. As a result, we were interested in determining whether serum markers of oxidative stress and/or damage might indicate potential adverse health effects.

Cytokines are low molecular weight, nonstructural proteins secreted by cells and having a major role in regulating inflammation and immunity. Although there have been reports in the literature investigating cytokine levels after the failure of knee and hip replacements [18–20, 36–38] as well as a result of acute muscle injury [39, 40], there have been no reports on perturbations in serum cytokine levels from embedded metal fragments such as those suffered in armed conflict. In this study, we assessed the serum levels of a variety of biomarkers linked to the induction and resolution of inflammation. Some of the measured cytokines showed little to no difference between the metal-implanted animals and control. For example, IL-1*β* levels in practically all cases were below the limit of detection of the assay method. Other markers, such as KC/GRO, showed no statistical differences between control and metal-implanted rats. Further, serum TNF-*α* levels were only significantly lower in the 12-month Al cohort, with no other statistically significant differences observed. However, there were significant differences in many of the other cytokines measured.

IFN-*γ*, a potent macrophage activator, was significantly elevated in the serum of most of the metal-implanted rats particularly at the 3-month time point. In most cases, levels revert to normal, but in the case of the Fe and Cu cohorts, they remain elevated for extended periods. The fact that Fe and Cu can catalyze the Fenton and Haber-Weiss reactions [41, 42] to produce reactive oxygen species may play a role in the enhanced elevation of IFN-*γ*, but this hypothesis requires further investigation.

Serum IL-5 levels were significantly higher in the Co, Fe, and Cu cohorts but only in the 12-month postimplantation groups. IL-5 has been shown to be elevated in synovial fluid from patients with failed Co/Cr/Mo metal-on-metal hip arthroplasties [36]. IL-5 has a variety of roles with B-cell differentiation being the most prominent. IL-5 levels are also elevated in serum from Ni-allergic individuals [43]; however, implantation of Ni pellets had no effect on IL-5 levels in the serum of the rats.

IL-6 is classified as a proinflammatory cytokine. However, depending upon the experimental situation, IL-6 has been ascribed to both pro- and anti-inflammatory properties [40, 43–45]. Some investigators have used the term “inflammation-responsive” as it does not directly induce inflammation and, in fact, is able to mediate anti-inflammatory responses [40, 46–49]. In our model, all of the implanted test metals, except for Ni, resulted in significantly elevated serum levels of IL-6 usually starting at the 3-month postimplantation time point, and in some case, serum levels of IL-6 were still elevated at the 12-month time point.

The other cytokines investigated (IL-4, IL-10, and IL-13) have all been identified as having anti-inflammatory properties and a role in the repair of muscle damage [40]. The patterns for these four cytokines are remarkably similar across most of the metal implantation groups with significantly higher serum levels in the 3- and 6-month time points. For some metals such as Co, Fe, and Cu, the levels remain elevated at the 12-month time point while for others (Al, Pb, and DU), the levels decrease to well below control values. Although beyond the scope of this project, it would be informative to extend the collection time points to cover the entire lifespan of the rat since many of the adverse health effects of embedded shrapnel from war wounds reported in the literature occur many years after the initial injury.

Taken together, these data indicate that, even in the absence of overt histopathological damage, embedded metal fragments can affect serum cytokine levels. Many, but not all, of the cytokines assessed were significantly elevated in serum. Somewhat surprisingly, only IFN-*γ* and IL-4 were significantly elevated at 1 month postimplantation: IFN-*γ* for Cu and Al and IL-4 for Pb. On the other hand, rats implanted with DU pellets showed significantly decreased levels of IFN-*γ*, IL-4, IL-6, IL-10, and IL-13 at the early time points before reaching significantly higher levels at the later experimental points, suggesting that DU inhibits cytokine expression shortly after implantation by an as yet unidentified mechanism.

Oxidative stress occurs when the production of free radicals overwhelms the cell's antioxidant defense systems. This can result in the oxidative damage of DNA, lipids, and proteins. This damage can potentially result in the induction of inflammation, genotoxicity, and carcinogenesis. Although information on embedded metal fragments is lacking, exposure to metals in a variety of experimental systems has been shown to result in the induction of oxidative stress [50–53]. Even though many metals have been shown to induce oxidative damage, Cu and Fe have received the most study because of their endogenous nature and ability to participate in the Fenton and Haber-Weiss reactions [41, 42]. Both Cu and Fe have been shown to generate reactive oxygen and nitrogen species resulting in oxidative damage that depletes cellular antioxidant defenses and adversely affects the ability of the cell to maintain energy homeostasis [54–56]. Depletion of cellular antioxidant defenses also leads to oxidative damage to proteins, lipids, and DNA [57–60].

C-reactive protein is a liver protein that has been identified as a biomarker of low-level inflammation. Previously published research has indicated that serum C-reactive protein levels correlate positively with Cu serum levels and negatively with selenium levels [61]. We did not see any significant effect of Cu implantation on C-reactive protein levels at any of the time points examined. At 3 months postimplantation, levels were significantly higher in the Ni and Fe cohorts. For the W, Co, Fe, and Cu groups, serum C-reactive protein levels tended to rise as the animal aged. Conversely, in the Al, Pb, and DU cohorts, the levels trended downward over time and at 12 months postimplantation were significantly lower than those in the control.

Metals have been shown to affect lipid peroxidation [62, 63] and can be loosely grouped into those that undergo redox-cycling reactions and those that are redox-inactive whose major effect is depleting the antioxidant glutathione [64]. Metals in the former category include Fe, Cu, and Co while those in the latter include Ni and Pb. Using the TBARS assay to assess lipid peroxidation in the serum of metal-implanted rats, we found that while lipid peroxidation generally tended to increase over the entire experimental period for all metals with both the Al and Pb 12-month groups demonstrating significantly elevated levels of lipid peroxidation over the control, there were significantly lower levels at the 6-month time point for Fe, Cu, Al, Pb, and DU groups. Although Pb and Al are considered redox-inactive, both have been shown to increase lipid peroxidation in a variety of experimental systems. In a rat model, oral exposure to Pb increased lipid peroxidation levels in blood plasma and salivary glands [65]. Pb exposure also induced lipid peroxidation in the blood, kidney, and liver [66] with the increase due to a decrease in glutathione and other cellular antioxidant defenses [67]. Al exposure also induced lipid peroxidation in a variety of organs [68]. In this case, it is believed that Al acts as a prooxidant and facilitates oxidative stress through superoxide- and endogenous Fe-associated mechanisms [69, 70]. Using a freshwater snail model and exposure to several heavy metals, Mnkandla et al. [71] showed that metal exposure could result, at least temporarily, in a decrease in lipid peroxidation by upregulating the expression of superoxide dismutase, catalase, and glutathione peroxidase, enzymes involved in the cellular antioxidant defense system. It is likely that a similar situation could be occurring in our study with an increase in antioxidant defenses at the 6-month time point followed by depletion or overwhelming of the defenses followed by an increase in lipid peroxidation. An investigation into the metal effects on the expression of the enzymatic antioxidant defenses is beyond the scope of this submission. However, in an earlier report, we showed that 4-hydroxynonenal-modified proteins in the area of the gastrocnemius where the metal pellets were implanted were significantly increased at 12 months postimplantation in the Fe, Cu, Pb, and DU groups suggesting that some, but not all, embedded metals can induce lipid peroxidation [72]. Since several of the test metals are considered “redox-inactive” and are capable of depleting glutathione, we also measured total glutathione levels in serum. Glutathione is the most abundant low molecular weight thiol in cells and plays a major role in antioxidant defense and maintenance of the cellular redox state [73–75]. In particular, it is a key player in the amelioration of metal-induced oxidative stress by maintaining redox balance as well as by chelating metals that contribute to oxidative damage [76]. Despite the important role glutathione plays in regulating metal-induced oxidative stress, we found no significant differences with any of the metals at the time points assessed. We also did not find any significant effect of metals on oxidatively damaged proteins assessed by the advanced oxidation protein products assay. In fact, there was only one value significantly different from the control and that was the 12-month DU group, and it was lower than the control.

## 5. Conclusions

Injuries with embedded metal fragments are an unfortunate consequence of armed conflicts. Technological advances in weapon systems and the insurgent use of improvised explosive devices mean that the types of metal found in these wounds are virtually unlimited. Standard surgical guidance is to leave embedded metals in place except under certain conditions in order to avoid the potential morbidity that extensive surgery can bring. Unfortunately, in many cases, the long-term toxicological properties of embedded metals are not known, further complicating treatment decisions. In this report, we expanded upon our earlier research investigating the health effects of embedded military-relevant metals and showed that the embedded metals can affect serum cytokine profiles. In particular, IFN-*γ*, IL-4, IL-5, IL-6, IL-10, and IL-13 all demonstrated metal-induced increases. While C-reactive protein and lipid peroxidation by-products were also affected by the embedded metals, there were no indications of oxidative protein damage and glutathione levels were also not affected. Taken together, the results described suggest that serum cytokine levels may prove useful in developing early detection profiles for identifying potential adverse health effects of various embedded metals.

## Data Availability

All data supporting the results described are provided within the manuscript.
